# Lameness Prevalence and Risk Factors in Large Dairy Farms in Upstate New York. Model Development for the Prediction of Claw Horn Disruption Lesions

**DOI:** 10.1371/journal.pone.0146718

**Published:** 2016-01-21

**Authors:** Carla Foditsch, Georgios Oikonomou, Vinícius Silva Machado, Marcela Luccas Bicalho, Erika Korzune Ganda, Svetlana Ferreira Lima, Rodolfo Rossi, Bruno Leonardo Ribeiro, Arieli Kussler, Rodrigo Carvalho Bicalho

**Affiliations:** 1 Department of Population Medicine and Diagnostic Sciences, College of Veterinary Medicine, Cornell University, Ithaca, NY, United States of America; 2 Department of Epidemiology and Population Health, Institute of Infection and Global Health, University of Liverpool, Cheshire, United Kingdom; The University of Melbourne, AUSTRALIA

## Abstract

The main objectives of this prospective cohort study were a) to describe lameness prevalence at drying off in large high producing New York State herds based on visual locomotion score (**VLS**) and identify potential cow and herd level risk factors, and b) to develop a model that will predict the probability of a cow developing claw horn disruption lesions (**CHDL**) in the subsequent lactation using cow level variables collected at drying off and/or available from farm management software. Data were collected from 23 large commercial dairy farms located in upstate New York. A total of 7,687 dry cows, that were less than 265 days in gestation, were enrolled in the study. Farms were visited between May 2012 and March 2013, and cows were assessed for body condition score (**BCS**) and VLS. Data on the CHDL events recorded by the farm employees were extracted from the Dairy-Comp 305 database, as well as information regarding the studied cows’ health events, milk production, and reproductive records throughout the previous and subsequent lactation period. Univariable analyses and mixed multivariable logistic regression models were used to analyse the data at the cow level. The overall average prevalence of lameness (VLS > 2) at drying off was 14%. Lactation group, previous CHDL, mature equivalent 305-d milk yield (ME305), season, BCS at drying off and sire PTA for strength were all significantly associated with lameness at the drying off (cow-level). Lameness at drying off was associated with CHDL incidence in the subsequent lactation, as well as lactation group, previous CHDL and ME305. These risk factors for CHDL in the subsequent lactation were included in our predictive model and adjusted predicted probabilities for CHDL were calculated for all studied cows. ROC analysis identified an optimum cut-off point for these probabilities and using this cut-off point we could predict CHDL incidence in the subsequent lactation with an overall specificity of 75% and sensitivity of 59%. Using this approach, we would have detected 33% of the studied population as being at risk, eventually identifying 59% of future CHDL cases. Our predictive model could help dairy producers focusing their efforts on CHDL reduction by implementing aggressive preventive measures for high risk cows.

## Introduction

Lameness, defined as abnormal gait and usually associated with painful lesions in the foot, is undeniably one of the costliest issues the modern dairy industry worldwide needs to address. The average cost per case (US$) of sole ulcer, digital dermatitis and foot rot was estimated at 216, 133 and 121, respectively [1], while lameness in general has been estimated to cost the UK cattle industry £54 million per year [2]. Additionally, lameness is a significant welfare issue because of its high prevalence in herds throughout the world and its debilitating effects [3, 4]. In New York State, the incidence of lameness (defined as a locomotion score of 3 or higher; 1–5 scale) within the first 70 days in milk varied from 27% to 54% [5]; in Wisconsin, Cook et al. (2003) reported an incidence of lameness (defined as a locomotion score of 3 or 4; 1–4 scale) of 21% in summer and 24% in winter. In the UK the prevalence of lameness appears to be on the rise, with earlier reports indicating prevalence of lameness (defined as a locomotion score of 3 or higher; 1–5 scale) below 25% [6–8] and more recent reports indicating prevalence above 35% (defined as a locomotion score of 2 or 3; 0–3 scale) [9].

Factors related to the cow’s environment have been associated with lameness. Lameness prevalence has been shown to be higher in freestall barns comparing to tie-stall barns [3, 10]. Stall and alley surfaces are associated with lameness risk as well. Studies in Wisconsin and in upper Midwest US dairies have shown that farms using mattress- based stalls have a higher prevalence of lameness than farms using sand-based stalls [3, 11] or deep bedded stalls [12, 13]. A recent European study associated housing and management with welfare indicators, including lameness [14]. M. de Vries et al. (2015) reported that prevalence of lameness was lower in Dutch dairy herds using mattresses or deep bedding compared with concrete. Several studies demonstrated that the use of rubber flooring is beneficial to dairy cows housed in freestall barns, decreasing the trauma and wear [15–18]. Heat stress, high stocking rates and inadequate feed bunk space per cow are also considered to be lameness risk factors, since they could lead in increased daily standing times [11, 19, 20].

Sole ulcers and white line disease (often studied together and reported as claw horn disruption lesions (**CHDL**)) have for long been reported to be the most prevalent diseases associated with lameness [21] representing over 65% of all lesions diagnosed in lame cows [5]. Our group has recently shown that subsequent lactation CHDL incidence can be predicted with an overall good accuracy using data collected at drying off (BCS and/or digital cushion thickness, age of the cow and previous lactation CHDL incidence) [22]. However, this study was conducted in one farm using data from 574 animals. Therefore, it was considered important that before such lameness prediction models can be effectively implemented on commercial dairy farms, their validation in a multi-farm study with a greater number of animals/ observations is warranted.

The dairy industry has experienced significant changes in the last decades. Between 2001 and 2009, the number of farms in the USA decreased from 97,460 to 65,000, while the number of animals per farm increased [23]. The USDA also reported in 2010 that the number of farms with 2,000 cattle or more increased from 12% to 30% between those years. Genetic improvement along with better management practices are reflected by higher productivity per cow. Milk production has increased 15%, from 165,332 in 2001 to 189,320 million pounds in 2009, whereas the number of dairy cows has grown only 1%, from 9.10 to 9.20 million [23]. Therefore, we focused the present study in large high producing farms.

The main objectives of this study were a) to describe lameness prevalence at drying off in large high producing New York State herds and identify potential cow and herd level risk factors, and b) to develop a model that will predict the probability of CHDL in the subsequent lactation using variables collected at drying off and/or available from farm management software.

## Materials and Methods

### Ethics statement

This study was carried out in strict accordance with the recommendations of The Animal Welfare Act of 1966 (AWA) (P.L. 89–544) and its amendments 1970 (P.L. 91–579); 1976 (P.L. 94–279), 1985 (P.L. 99–198) which regulate the transportation, purchase, care, and treatment of animals used in research. The research protocol was reviewed and approved by the Institutional Animal Care and use Committee of Cornell University (Protocol number: 2008–0096). All farm owners were in agreement with the observational study to be performed in their properties.

### Farms and management

Data were collected from twenty three large commercial dairy farms located in upstate New York. These farms were selected either because of their relationship with the Ambulatory and Production Medicine Clinic at Cornell University (Ithaca, NY, USA) or because they were recommended by veterinarians and industry representatives. To be included in the study, all farms had to use Dairy-Comp 305 (Valley Ag Software, Tulare, CA) as management software, be enrolled in the Dairy Herd Improvement Association program (Dairy One, Ithaca, NY, USA) and have a good history of record keeping and disease diagnosis. Most of these farms were clients of the Ambulatory and Production Medicine clinic at Cornell University and therefore their employees had been trained by Cornell clinicians in foot trimming and lesion identification and recording. Farmers that were not clients of the Ambulatory and Production Medicine clinic were interviewed before enrolment and were only included in the study if they were able to provide enough evidence showing that their employees responsible for foot trimming were adequately trained and that they were consistent in recording CHDL. The approximate milking herd size of the study farms ranged between 600 and 4,000 cows. In all study dairy farms, cows were housed in free-stall barns. Four different types of stalls were used: 1) deep stalls bedded with sand, 2) deep stalls bedded with organic bedding material (recycled composted manure solids or recycled paper pulp), 3) concrete stalls covered with rubber mattresses and bedded with organic bedding material, or 4) concrete stalls covered with water beds bedded with organic bedding material. The feed alleys had either grooved concrete flooring or rubber flooring. The cows’ diets were formulated to meet or exceed the National Research Council (2001) nutrient requirements for lactating or dry Holstein cows weighing 650 kg and producing 45 kg of 3.5% fat corrected milk. Non-lactating cows were grouped on the basis of days from parturition: a far-off group (> 30 days from parturition) and a close-up group (≤ 30 from parturition) in most of the farms.

### Study design and data collection

A prospective cohort study was conducted and 7,687 cows were enrolled from May 2012 to March 2013. Researchers visited the participating farms and at each visit BCS and visual locomotion score (VLS) were assessed by one single investigator for all dry cows that were less than 265 days in gestation. Body condition score was scored on a scale from 1 to 5 (1 = emaciated, 5 = obese, scored in 0.25-point intervals) [24]. Visual locomotion score (VLS) was recorded on a scale from 1–5 (1 = normal, 5 = extremely (non-weight-bearing) lame, in 1-point intervals) [5].

Data on the claw horn disruption lesion (CHDL) events recorded by the farm employees were extracted from the Dairy-Comp 305 database. Events that occurred in the previous and in the subsequent lactations including sole ulcers and white line disease were imported into DC305 and subsequently extracted and grouped into the variable CHDL (0 = unaffected and 1 = affected). Infectious diseases such as digital dermatitis, interdigital phlegmon, and interdigital dermatitis were excluded from the database.

The Dairy-Comp 305 database was also used to obtain data regarding the studied cows’ health events, milk production, and reproductive records throughout the lactation period. Moreover, during the visits, information regarding average stocking rate of lactating and dry cows’ pens, feed bunk space available per cow, the type of bedding, and flooring used in the lactating and dry cow barns, was collected.

Information regarding each enrolled cow’s sire genetic evaluation was also obtained from Dairy-Comp 305 database. Available information included: predicted transmitting ability (PTA) for milk yield, fat yield, fat percentage, protein yield, protein percentage, and standardized PTA for strength, rump angle, thurl width, rear udder height, rear udder width, udder depth, fore udder attachment, teat placement, rear legs, foot angle and stature.

### Statistical analyses

To facilitate data analysis and interpretation of results the following variables were created: BCS group at drying off (**BCSG**): BCSG 1 if BCS < 3; BCSG 2 if 3 ≤ BCS < 3.5; BCSG 3 if BCS ≥ 3.5. Lactation group (**LACTG**): LACTG 1 if lactation = 1; LACTG 2 if lactation = 2; LACTG 3 if lactation ≥ 3. Lameness incidence at drying off (**LAMEDRY**): LAMEDRY 0 if VLS ≤ 2; LAMEDRY 1 if VLS > 2. Severe lameness incidence at drying off (**SEVLAME**): SEVLAME 0 if VLS ≤ 3; SEVLAME 1 if VLS > 3.

Using lameness related information from Dairy-Comp 305 and focusing on sole ulcers and white line disease incidence, the following variables were also created: Previous lactation CHDL incidence (PCHDL) (0/1); Subsequent lactation CHDL incidence (CHDL) (0/1); Subsequent lactation CHDL incidence during the first 100 days of lactation (CHDL100) (0/1).

Microsoft Excel (Microsoft, Redmond, MA), SAS 9.3 and JMP Pro11 (SAS Institute Inc., Cary, NC) were used to create the final project database and to perform statistical analysis of the data.

Associations of mean farm lameness (VLS > 2) and severe lameness (VLS > 3) incidence at drying off and mean farm previous and subsequent lactation CHDL incidence with herd level continuous variables were examined using multivariate analysis in JMP Pro11. Herd level variables that were considered were: stocking rates for dry and lactating cows (SRDC, SRLC), and feed bunk space for dry and lactating cows (FBDC, FBLC).

Associations of mean farm lameness (VLS > 2) and severe lameness (VLS > 3) incidence at drying off and mean farm previous and subsequent lactation CHDL incidence with stall or flooring type (categorical variables) were examined using one-way ANOVA in JMP Pro11 and graphs were built with GraphPad Prism (GraphPad Software, Inc., La Jolla, CA).

The following variables were examined for associations with cow lameness (VLS > 2) at drying off in a series of univariable analyses: BCSG, LACTG, mature equivalent 305-d milk yield (ME305), previous lactation disease incidence (mastitis, retained placenta, metritis, displaced abomasum), PCHDL, and all sires’ PTAs. Variables with a P value ≤ 0.25 were entered in a mixed multivariable logistic regression model with cow lameness (VLS > 2) at drying off as the outcome variable. Variables were removed from the model manually in a stepwise manner and only variables with *P* < 0.05 were kept in the final model. All possible 2-way interactions between the independent variables were added to the model. Farm was included in the model as a random effect to account for within farm clustering.

The variables BCSG, LACTG, ME305, previous lactation disease incidence (mastitis, RP, metritis, DA), PCHDL, LAMEDRY, and all sires’ PTAs, were examined for associations with CHDL incidence in the subsequent lactation in a series of univariable analyses. Variables with a *P* ≤ 0.25 were fitted as explanatory variables in a mixed multivariable logistic regression model with CHDL incidence in the subsequent lactation as the outcome variable. Variables were removed from the model manually and in a stepwise manner at a value of *P* > 0.05. All possible 2-way interactions between the independent variables were added to the model and Farm was included as a random effect. Both mixed multivariable logistic regression models were built in SAS 9.3 using the GLIMMIX procedure. The above described logistic regression model (with CHDL incidence in the subsequent lactation as the outcome variable) was used to calculate the predicted probability of developing a CHDL in the subsequent lactation for each cow. To identify the optimum cut off point that would more accurately categorize cows as high or low risk for development of CHDL in the subsequent lactation, a receiver operator characteristic (**ROC**) analysis was conducted in Medcalc version 13.2.2. (MedCalc Software, Ostend, Belgium) using actual CHDL incidence in the subsequent lactation as the gold standard. Cows were also categorized in quartiles based on this predicted probability.

## Results

**Fig 1:**
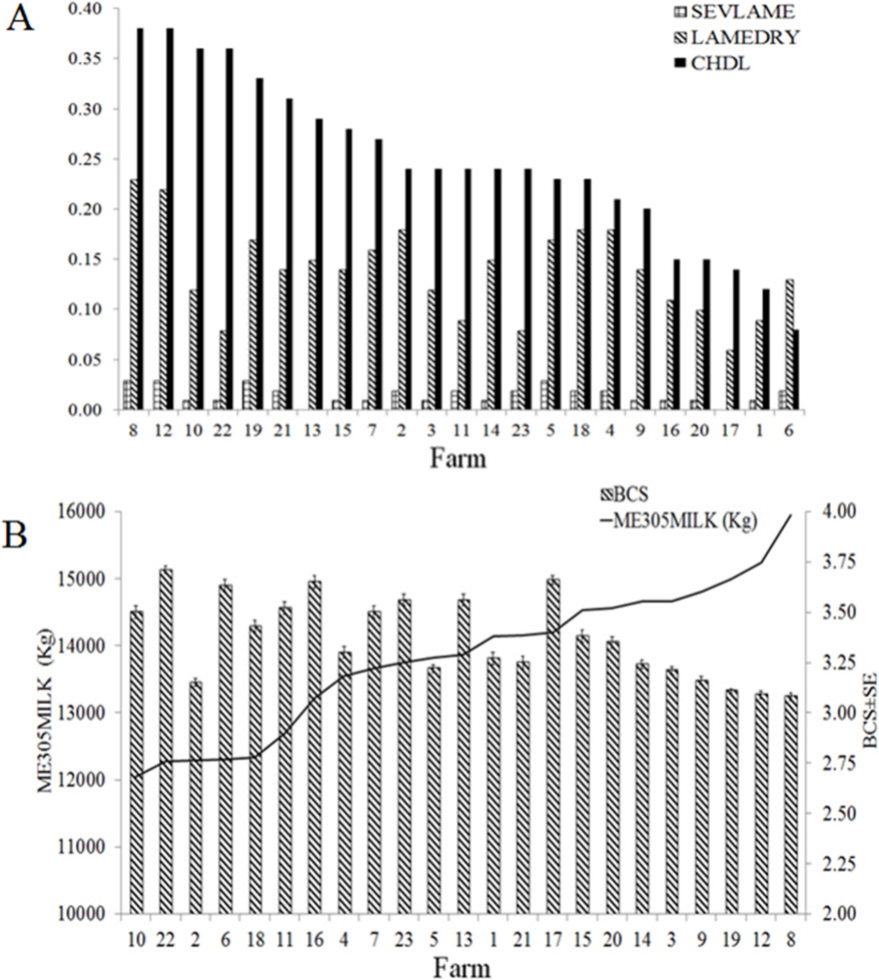
Descriptive statistics for enrolled farms. (A) Dry cows’ severe lameness prevalence (visual locomotion score > 3; SEVLAME); dry cows’ lameness prevalence (visual locomotion score > 2; LAMEDRY); claw horn disruption lesion incidence in the subsequent lactation to our visit (CHDL). (B) Mean (with standard error) mature equivalent 305-d milk yield in kg (ME305MILK); dry cows’ mean (with standard error) body condition score (BCS).

Descriptive statistics for each one of the 23 farms that were included in this study are presented in Fig 1. The average prevalence of lameness (visual locomotion score (VLS) > 2) at drying off for all the 23 farms was 14% (ranging from 6 to 23%), the mean prevalence of severe lameness (VLS > 3) was 2% (0–3%), the average incidence of claw horn disruption lesions (CHDL) in the subsequent lactation was 25% (8–38%), the mean body condition score at drying off was approximately 3.5 and the mean mature equivalent 305-d milk yield (ME305) was 13,814 ± 226 (mean ± SE).

**Fig 2:**
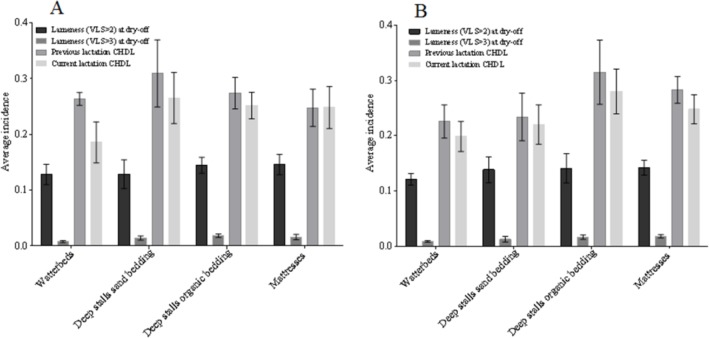
Lameness, severe lameness at dry-off and claw horn disruption lesion (CHDL) by different stall bedding type. Mean farm lameness (visual locomotion score (VLS) > 2) and severe lameness (VLS > 3) prevalence at drying off and mean farm previous and subsequent lactation claw horn disruption lesion (CHDL) incidence by different stall bedding type for dry (A) and lactating cows (B). Error bars represent standard error of the mean.

**Fig 3:**
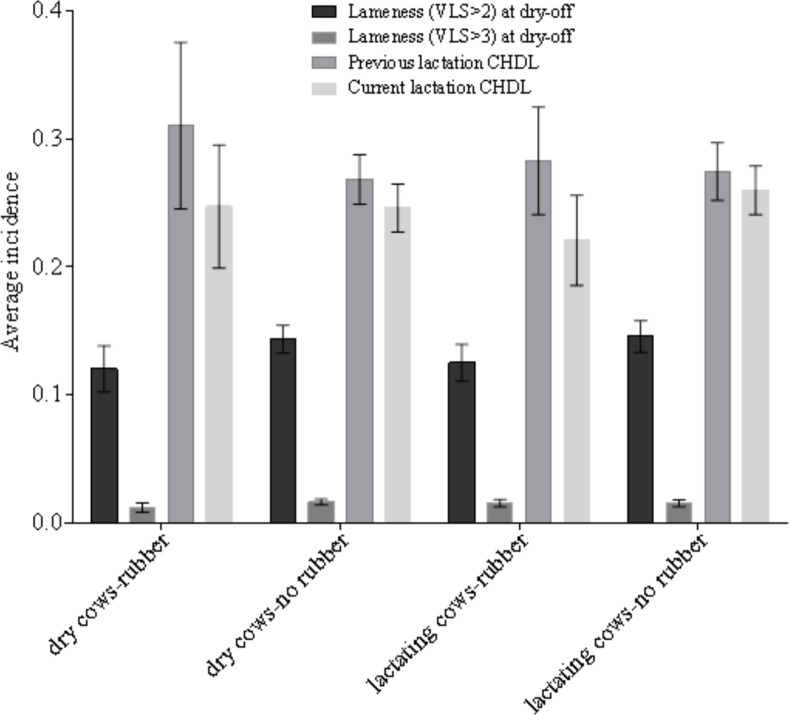
Lameness, severe lameness at dry-off and claw horn disruption lesion (CHDL) by rubber floor use. Average farm lameness (visual locomotion score (VLS) > 2) and severe lameness (VLS > 3) prevalence at drying off and average farm previous and subsequent lactation claw horn disruption lesion (CHDL) incidence for farms that were or were not using rubber flooring in the dry cows pens and for farms that were or were not using rubber flooring in the lactating cows pens. Error bars represent standard error of the mean.

Mean farm lameness (VLS > 2) and severe lameness (VLS > 3) incidence at drying off and mean farm previous and subsequent lactation CHDL incidence by different stall bedding type for dry and lactating cows are presented in Fig 2. Even though certain numerical differences were observed, none of them were statistically significant. Farms using deep sand bedding or water beds had numerically lower incidence of CHDL in both studied lactations. Average farm lameness (VLS > 2) and severe lameness (VLS > 3) incidence at drying off and mean farm previous and subsequent lactation CHDL incidence for farms that were or were not using rubber flooring in the dry cows pens and for farms that were or were not using rubber flooring in the lactating cows pens are presented in Fig 3. Differences were not statistically significant. Results presented in Figs 2 and 3 were obtained by one-way ANOVA in JMP Pro11.

**Fig 4:**
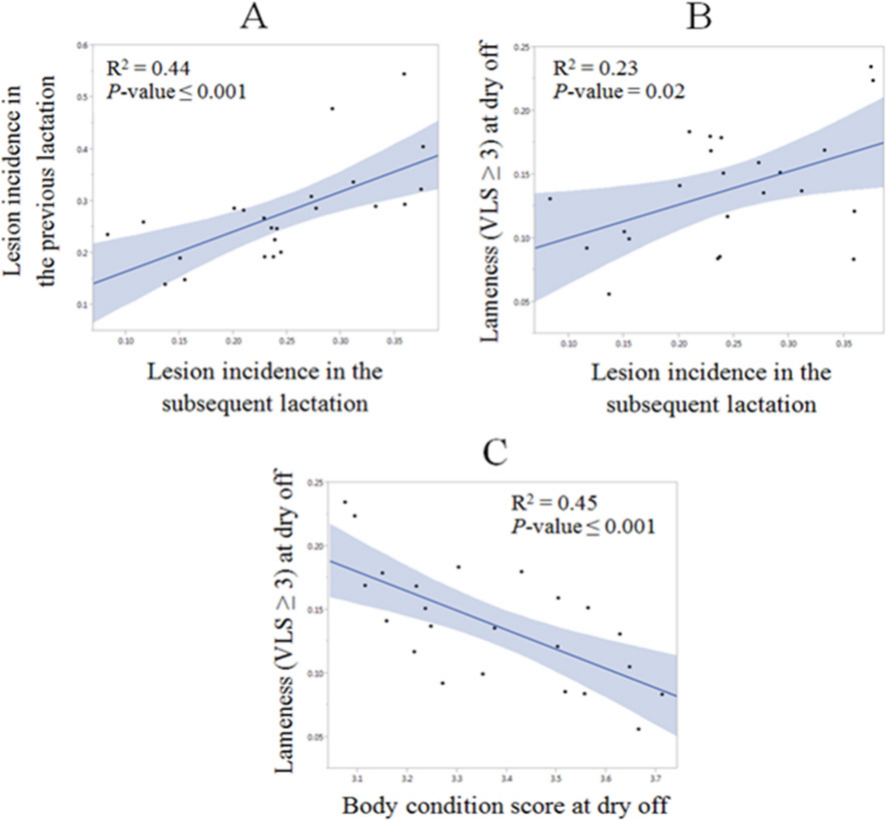
Linear regression scatter plots. Linear regression scatter plots (fitted lines with confidence intervals in grey) indicating (A) the positive association of the farm level incidence of claw horn disruption lesions (CHDL) in the previous lactation (Y axis) and the farm level incidence of CHDL in the subsequent lactation (X axis); (B) the positive association of the prevalence of lameness (visual locomotion score > 2) at drying off (Y axis) and the farm level incidence of CHDL in the subsequent lactation (X axis); (C) the negative association of the prevalence of lameness (visual locomotion score > 2) at drying off (Y axis) and the body condition score at drying off (X axis).

Linear regression scatter plots in Fig 4 indicate a) the positive association between the farm level incidence of CHDL in the previous lactation and the farm level incidence of CHDL in the subsequent lactation; b) the positive association between the prevalence of lameness (VLS > 2) at drying off and the farm level incidence of CHDL in the subsequent lactation; c) the negative association between the prevalence of lameness (VLS > 2) at drying off and BCS at drying off. All three associations presented were statistically significant (*P* ≤ 0.02). Results presented in Fig 4 were obtained by multivariate analysis in JMP Pro11.

Variables significantly associated with lameness prevalence at drying off (cow-level) were season, BCSG, PCHDL, LACTG, ME305, and sire’s PTA for strength. Explanatory variables retained in the logistic regression model with least squares means, 95% confidence intervals and *P* values are presented in Table 1 (except for the continuous variables ME305 and sire’s PTA for strength). None of the 2-way interactions between the independent variables were significant and therefore not retained in the final model. Thinner cows at dry off, specifically BCS ≤ 2.75, older cows (3^rd^ or greater than 3^rd^ lactation), or cows that were affected by CHDL during the previous lactation had higher adjusted lameness prevalence at drying off compared to cows with BCS ≥ 3.0, younger cows or cows that were not affected by CHDL before. Summer was the season with the highest adjusted mean lameness prevalence, followed by winter, autumn and spring. High milk yield (ME305 milk) in the previous lactation was associated with significantly decreased risk of lameness at drying off (fixed effect estimate = -0.00004, *P* < 0. 01). Sires’ PTA for strength was positively associated with lameness at drying off (fixed effect estimate = 0.1019, *P* = 0.01).

**Table 1:** Explanatory categorical variables retained in the logistic regression model with dry cows’ lameness prevalence (0 = visual locomotion score ≤ 2, 1 = visual locomotion score > 2) as an outcome variable. Least squares means with 95% confidence intervals (**CI**) and *P* values are presented.

		Mean (95% CI)	*P* value
SEASON	1	0.18 (0.14–0.24)	< 0.01
	2	0.10 (0.06–0.16)	
	3	0.21 (0.18–0.24)	
	4	0.16 (0.13–0.19)	
BCSG	1	0.25 (0.20–0.30)	< 0.01
	2	0.15 (0.12–0.18)	
	3	0.10 (0.08–0.13)	
PCHDL	0	0.09 (0.08–0.12)	< 0.01
	1	0.25 (0.21–0.30)	
Lactation group	1	0.09 (0.07–0.11)	< 0.01
	2	0.17 (0.14–0.21)	
	3	0.24 (0.20–0.29)	

SEASON: Season at scoring (1 = winter, 2 = spring, 3 = summer, 4 = autumn); BCSG: Body condition score at drying off (1 if BCS < 3; 2 if 3 ≤ BCS < 3.5; 3 if BCS ≥ 3.5); PCHDL: Previous lactation claw horn disruption lesion incidence (0/1); Lactation group (1 if lactation = 1; 2 if lactation = 2; 3 if lactation ≥ 3)

Variables significantly associated with CHDL incidence in the subsequent lactation were: LAMEDRY, LACTG, PCHDL, and ME305. Explanatory variables retained in the logistic regression model with least squares means, 95% confidence intervals and *P-*values are presented in Table 2 (except for the continuous variable ME305). None of the 2-way interactions between the independent variables were significant and therefore not retained in the final model. Cows that were found to be lame at drying off, older cows and cows that were affected with CHDL in the previous lactation had a higher association with CHDL incidence in the subsequent lactation. High milk yield (ME305 milk) in the previous lactation was associated with significantly increased risk of CHDL incidence in the subsequent lactation (fixed effect estimate = 0.00002, *P < 0*.*01*).

**Table 2:** Explanatory categorical variables significantly associated with claw horn disruption lesion in the subsequent lactation. Least squares means with 95% confidence intervals (**CI**) and *P* values are presented.

		Mean (95% CI)	*P* value
Dry cow lameness (VLS > 2)	0	0.26 (0.23–0.31)	< 0.01
	1	0.38 (0.33–0.44)	
Lactation group	1	0.24 (0.20–0.28)	< 0.01
	2	0.34 (0.30–0.39)	
	3	0.39 (0.34–0.44)	
PCHDL	0	0.22 (0.18–0.25)	< 0.01
	1	0.44 (0.39–0.50)	

Lactation group: 1 if lactation = 1; 2 if lactation = 2; 3 if lactation ≥ 3; PCHDL: Previous lactation claw horn disruption lesion incidence (0/1)

**Fig 5:**
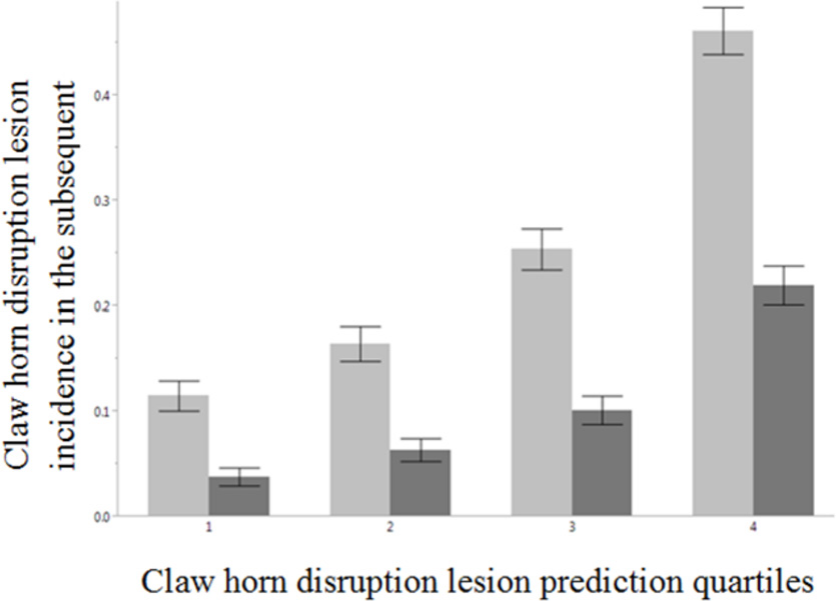
Claw horn disruption lesion (CHDL) incidence in the subsequent lactation by CHDL prediction quartile. The bars represent the mean CHDL incidence in the subsequent lactation (light gray) and mean CHDL incidence restricted to the first 100 days of the subsequent lactation (dark gray). Error bars indicate the 95% confidence interval.

Claw horn disruption lesion incidence in the subsequent lactation by CHDL prediction quartile is presented in Fig 5. The mean CHDL incidence in the subsequent lactation increased from the 1^st^ to the 4^th^ prediction quartiles.

**Fig 6:**
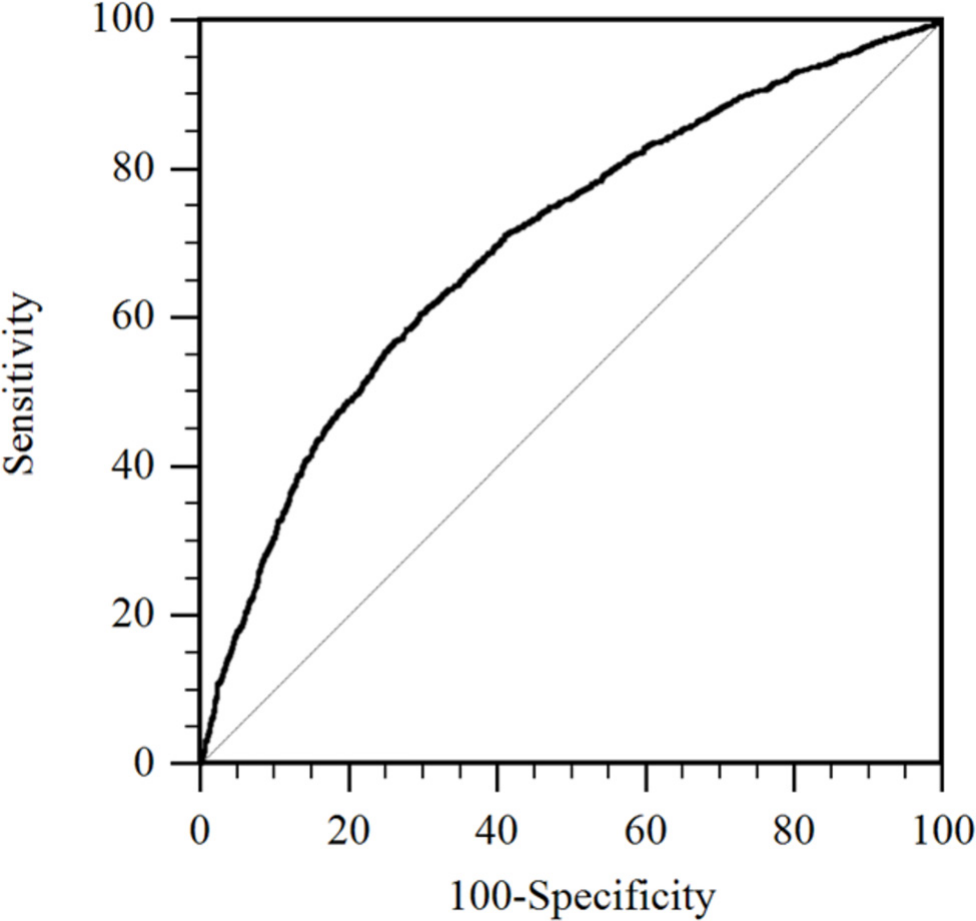
Receiver operating characteristic curve. ROC curve that identified the optimal cut off point for the predicted probability values obtained by the logistic regression model that had subsequent lactation claw horn disruption lesion (CHDL) as an outcome. The gold standard in this analysis was CHDL incidence.

**Fig 7:**
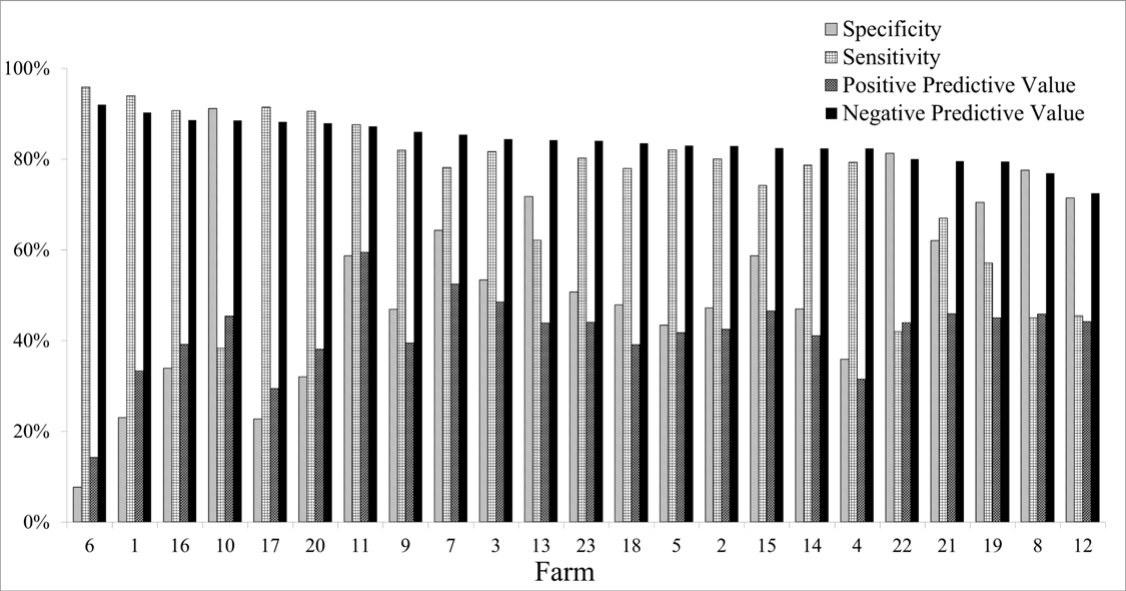
Specificity, Sensitivity, Positive Predictive Value and Negative Predictive Value. Bars for each farm, using the predicted probability threshold suggested by ROC analysis and claw horn disruption lesion incidence in the subsequent lactation as the gold standard.

Receiver operating characteristic curve was used to identify the optimal cut off point for the predicted probability values that were obtained from the logistic regression model that had subsequent lactation CHDL as an outcome (Fig 6). The gold standard in this analysis was the actual incidence of CHDL. A predicted probability greater than 0.27 was identified as the optimal cut off point that was then used to classify cows as high or low risk for development of CHDL in the subsequent lactation. Specificity, sensitivity, positive predictive value and negative predictive value for each farm are presented in Fig 7. Using 0.27 as a cut off value, the overall specificity, sensitivity, positive predictive value and negative predictive value obtained were 75%, 59%, 44% and 85%, respectively.

**Fig 8:**
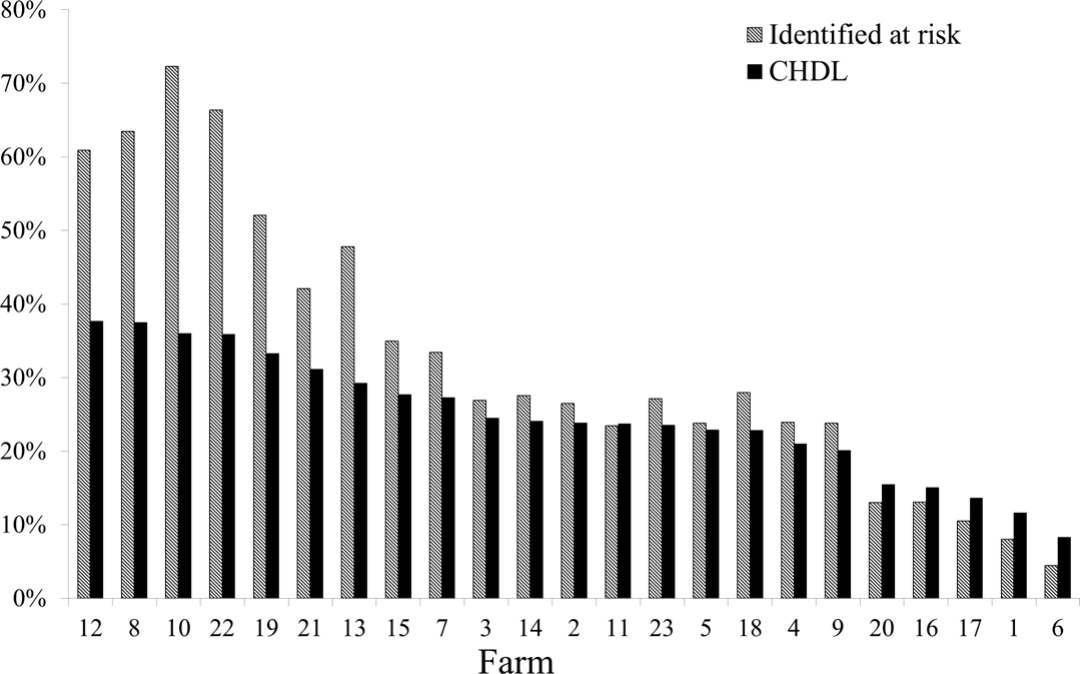
Percentage of cows identified as being at risk and percentage of cows that developed CHDL in the subsequent lactation. Percentage of cows identified as being at risk to develop claw horn disruption lesions (CHDL) in the subsequent lactation by the logistic regression model (using the cut off value derived by ROC analysis) and percentage of cows that developed CHDL in the subsequent lactation that were characterized as being at risk by the logistic regression model (using the cut off value derived by ROC analysis).

The percentage of cows identified by the logistic regression model (using the cut off value derived by ROC analysis) as being at risk to develop CHDL in the subsequent lactation is presented in Fig 8. The percentage of cows that actually developed CHDL in the subsequent lactation and were also identified as being at risk by the model is also shown in Fig 8. Overall, and using this prediction model, we would have detected 33% of the studied population as being at risk, eventually identifying 59% of future CHDL cases.

## Discussion

Lameness prevalence at drying off in large high producing New York State herds (average of 14% for VLS > 2 and 2% for VLS > 3) was similar to the prevalence described by Vergara et al. (2014) [25], who reported a mean prevalence of 11.2% (ranging from 8.7 to 13.4%) for dry cows in New York and Wisconsin. Lameness prevalence, assessed in the first 70 DIM, ranged between 26.5 to 54.2% of all cows in the 5 dairy farms evaluated in upstate New York by Bicalho et al. (2007). Keyserlingk et al. (2012) [26] reported a prevalence of clinical lameness of approximately 30% in British Columbia (BC), and in California (CA), and about 55% in northeastern United States (NE-US); prevalence of severe lameness averaged 4% in CA and approximately 8% in both BC and NE-US. Similar estimates were reported from studies conducted in the UK [9] and in China [27]. However, those studies were conducted on lactating cows, which are expected to have higher lameness prevalence comparing to dry cows since calving alone is a risk factor for lameness [28] while lactating cows are also experiencing negative energy balance and metabolic stress which can also predispose them to lameness [29]. The incidence of CHDL in the previous lactation as well as the prevalence of lameness (VLS > 2) at drying off were positively associated with incidence of CHDL in the subsequent lactation. This is in agreement with data presented by Hirst et al. (2002) [30], who observed that cows affected whith CHDL in the first lactation had a 3.2 hazard ratio for second lactation lameness caused by CHDL. This is also in agreement with findings from other studies [31, 32], who reported that the incidence of sole ulcer, and white line disease in the previous lactation was a strong predictor of future lactation incidence of the same lesions.

A statistically significant negative association between the prevalence of lameness (VLS > 2) at drying off and BCS at drying off was observed in our study. Body condition score is positively associated with the digital cushion thickness [33], which is composed of adipose tissue and plays an important function of reducing compression of corium tissue underneath the cushion [34, 35]. Therefore, the reduction in the digital cushion thickness caused by the fat mobilization may lead to higher CHDL incidence. Machado et al. (2010) [36] confirmed that low BCS and low digital cushion thickness at drying off were significant risk factors for CHDL during the subsequent lactation. Green et al. (2014) supported the hypothesis that low BCS contributes to the development of CHDL in a prospective longitudinal study. Additionally, lameness per se has been associated with body condition losses due to the altered resting and feeding behavior [37, 38, 39].

Older cows are more likely to be thinner and have a higher prevalence of lameness [22]. Our results were similar to results from previous studies, which associated increasing parity with higher incidence of CHDL at drying off [36], with lameness prevalence during lactation [5] and with higher risk of CHDL [40, 41, 42]. The weakening and decreased elasticity of the connective tissue between the hoof horn and the bone of the third phalanx close to calving [28] may be a possible explanation for this observation as greater parity cows have gone through this high risk period more times.

Bicalho et al. (2008) [43] indicated that high yield in early lactation was a risk factor for lameness. Furthermore, Amory et al. (2008) [44] found that high yielding cows were more likely to develop non-infectious causes of lameness. Similarly, our analysis showed that high milk production was positively associated with CHDL in the subsequent lactation. This is likely due to the extensive body condition loss and to metabolic disorders faced by high producing cows [45, 46]. In a longitudinal study, highest yielding cows were more likely to be thin (BCS < 2.5) and had a higher risk of becoming lame with CHDL [32]. On the other hand, high milk yield was negatively associated with lameness (VLS > 2) at drying off.

Sire PTA for strength increased the odds of lameness at drying off in the present study. A larger study from our group focused on the associations between sire predicted transmitting ability (PTA) for conformation and yield traits and the incidence of foot lesions [31]. Several associations were found to be significant, including sire PTA for milk and protein yield with the incidence of sole ulcers, white line disease and digital dermatitis and incidence of sole ulcer and white line disease, respectively. Besides those, sire PTA for several conformation traits were associated with sole ulcers, white line disease and digital dermatitis incidence. However, the Oikonomou et al. (2013) study was based on data collected on one large dairy herd and this could be the reason for the different results comparing to the described here study. Studies conducted in better controlled environments (e.g., one farm versus multiple farms) stand better chances of identifying associations that are due to genetics.

Herd level factors associated with increased daily standing times include season, poor stall design and comfort, overstocking and prolonged milking times [13, 47]. In our study, cows scored in the summer had a higher association with lameness (VLS > 2) at drying off. Mean farm lameness prevalence at drying off was numerically lower in farms that were using concrete stalls covered with waterbeds or deep stalls bedded with sand. However, the differences between types of stalls were not statistically significant. When the herd level factors were analyzed, farm was considered to be the study unit and this decreases the statistical power of this part of our study.

One of the aims of this study was the development of a model to predict the probability of CHDL in the subsequent lactation using variables collected at drying off and/or available from farm management software. Lameness prevalence at drying off, lactation group, previous lactation CHDL and ME305 were included in the prediction model, which had an overall specificity of 75% and sensitivity of 59%. Depending on the farm, the positive predictive value ranged between 14 and 59%, and the negative predictive value was from 72 to 92%. Machado et al. (2011) included BCS, age, and CHDL at cessation of lactation from 574 cows in a logistic regression model and were able to predict subsequent lactation CHDL incidence with good accuracy, approximately 75%. Data from only one farm was included in that study, whereas 23 different farms were enrolled in the current study.

Despite the fact that the prediction capacity of our model was moderate compared to previous work from our group, it could still be a useful tool for dairy farmers. Visual locomotion scores can easily be collected at drying off and entered in the Dairy Comp database. Bicalho et al. (2007) evaluated the performance of visual locomotion scoring (VLS) and Stepmetrix locomotion scoring (SLS) in detecting painful digit lesions. The authors concluded that when performed by trained veterinarians (which was the case in the study described here), VLS performed better than SLS in detecting painful lesions. Admittedly, VLS is a relatively subjective evaluation but is currently the gold standard in lameness detection and can provide with very useful information regarding herd level lameness prevalence. We show here that VLS can also be used to predict future CHDL incidence. Furthermore, parity, previous lactation CHDL and ME305 are already recorded by farmers. Separating higher risk cows in a pen closer to the milking parlor, providing better comfort to them and evaluating (not necessarily trim) their hooves more frequently are some of the strategies that farmers can implement to prevent CHDL, using our model to target cows at a higher risk of becoming lame in the subsequent lactation. Milking this group of cows two times a day instead of three could also be beneficial [48]. Culling decisions can also be made based on information provided by the same model.

## Conclusion

The prospective cohort study described here shows that the average prevalence of lameness (visual locomotion score > 2) at drying off was 14% for 23 high productive dairy herds in upstate NY. Lactation group, previous CHDL and ME305, together with season, BCS at drying off and sire PTA for strength were all associated with lameness at the drying off. Lameness at drying off was correlated with CHDL in the subsequent lactation, as well as lactation group, previous CHDL and ME305. Our predictive model could potentially help dairy farmers focusing their efforts on CHDL reduction by implementing aggressive preventive measures for high risk cows.
